# 502. Epidemiology and Outcomes of Broad-Spectrum Antibiotic De-escalation in Patients with Suspected Sepsis in US Hospitals

**DOI:** 10.1093/ofid/ofae631.154

**Published:** 2025-01-29

**Authors:** Kai-Qian Kam, Tom Chen, Sameer S Kadri, Alexander Lawandi, Christina Yek, Morgan Walker, Sarah Warner, David Fram, Huai-Chun Chen, Claire N Shappell, Laura DelloStritto, Robert Jin, Michael Klompas, Chanu Rhee

**Affiliations:** Harvard Pilgrim Health Care Institute, KK Women's and Children's Hospital Singapore, Duke-NUS Medical School Singapore, Singapore, Singapore; Harvard Medical School / Harvard Pilgrim Health Care Institute, Boston, Massachusetts; National Institutes of Health Clinical Center, Bethesda, Maryland; 1. Critical Care Medicine Department, Clinical Center, National Institutes of Health, Bethesda, MD, Montreal, Quebec, Canada; National Institute of Allergy and Infectious Diseases, Bethesda, Maryland; Critical Care Medicine Department, Clinical Center, National Institutes of Health, Critical Care Medicine Branch, National Heart Lung and Blood Institute, Bethesda, MD; Critical Care Medicine, National Institutes of Health Clinical Center, Bethesda, Maryland; Commonwealth Informatics, Waltham, MA; Commonwealth Informatics, Waltham, MA; Brigham and Women's Hospital, Boston, Massachusetts; Harvard Medical School / Harvard Pilgrim Health Care Institute, Boston, Massachusetts; Harvard Medical School / Harvard Pilgrim Health Care Institute, Boston, Massachusetts; Harvard Medical School and Harvard Pilgrim Health Care Institute, Boston, Massachusetts; Brigham and Women's Hospital / Harvard Medical School, Boston, MA

## Abstract

**Background:**

Guidelines recommend de-escalation of broad-spectrum antibiotics initiated for suspected sepsis based on clinical status and microbiological results after 48-72 hours, yet real world patterns of de-escalation remain unknown. We aimed to evaluate the frequency, hospital-level variation, predictors, and clinical outcomes of antibiotic de-escalation in suspected sepsis.

**Figure:**
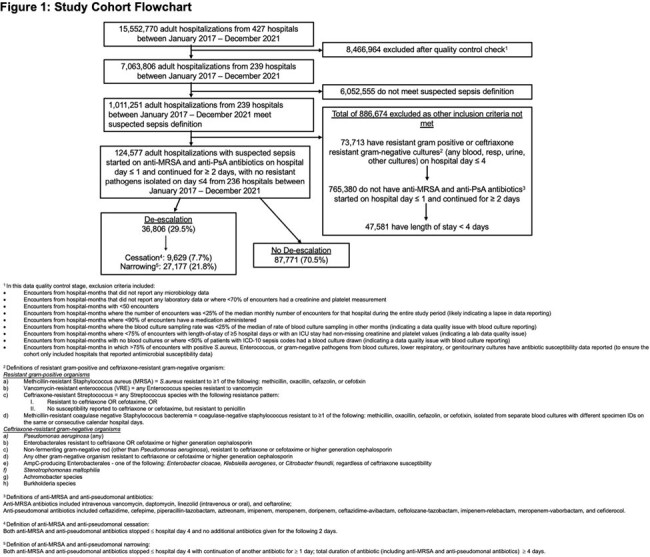

**Methods:**

We used the PINC AI^TM^ Healthcare Database to retrospectively analyze adults admitted to 236 US hospitals between 2017-2021 with suspected sepsis (blood culture and lactate drawn) and minimal 4-day stay who received ≥2 days of empiric anti-MRSA and anti-pseudomonal antibiotics on admission, in the absence of any beta-lactam resistant gram-positive or ceftriaxone-resistant gram-negative organisms in the clinical cultures by day 4.De-escalation was defined as cessation or switching to narrower spectrum intravenous or oral agents by day 4. We utilized a multivariate logistic regression model with 82 covariates to predict the likelihood of de-escalation; the probabilities from this model were then used for propensity-score matching.

**Figure:**
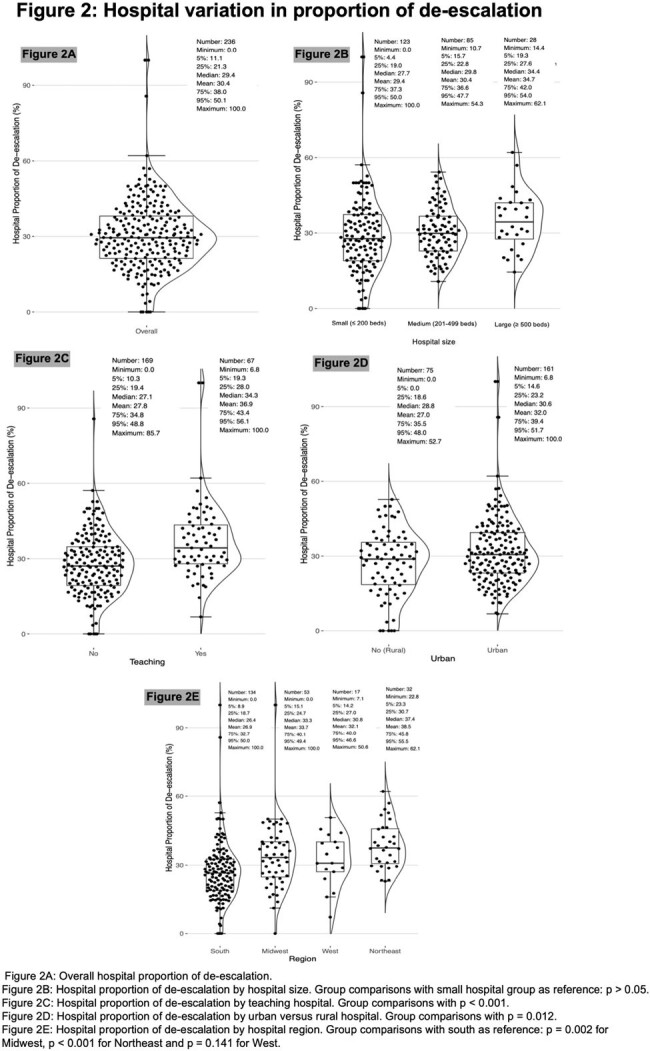

**Results:**

Among 124,577 patients who met inclusion criteria, 36,806 (29.5%) had antibiotics de-escalated (21.8% narrowed, 7.7% stopped) (Figure 1). The median hospital-level de-escalation rate was 29.4% (IQR: 21.3-38.0%) and varied across hospital types (Figure 2). Predictors of de-escalation included clinical indicators of less severe disease (especially on days 3-4), positive cultures, and negative/absent MRSA nasal swabs (Figure 3). De-escalation was also associated with medium, large or teaching hospitals in the Northeast or Midwest region. On propensity-matched analysis (effective sample size of 32,964 and 36,803 in control and treated groups, respectively), de-escalation was associated with lower rates of acute kidney injury (OR 0.91, 95% CI 0.85-0.96), inpatient mortality (OR 0.89, 95% CI 0.83-0.96), and a trend towards fewer *C.difficile* infections (OR 0.84, 95% CI 0.71-1.01) (Table 1).

**Figure:**
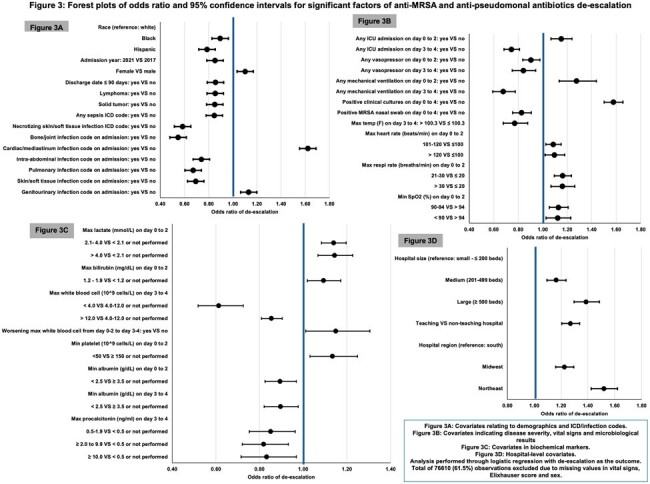

**Conclusion:**

In this large US cohort, antibiotic de-escalation in patients with suspected sepsis was infrequent and variable across hospitals. De-escalation was influenced by clinical and microbiologic factors and associated with lower risk for adverse outcomes.

**Figure:**
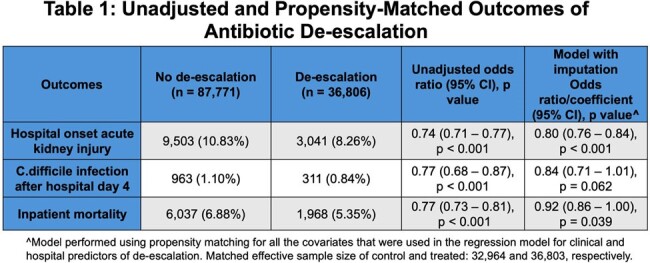

**Disclosures:**

**Michael Klompas, MD, MPH**, AHRQ: Grant/Research Support|CDC: Grant/Research Support|UpToDate: Royalties

